# Clinical Efficacy Comparison Between Brush-and-Floss and Brush-and-Rinse Daily Oral Hygiene Regimens on Plaque and Gingivitis in a Six-Week Randomized Clinical Model

**DOI:** 10.7759/cureus.110519

**Published:** 2026-06-09

**Authors:** Jeffery L Milleman, Reinhard Schuller, Kimberly R Milleman, Gregori M Kurtzman

**Affiliations:** 1 Clinical Research, Salus Research, Fort Wayne, USA; 2 Pharmacology and Therapeutics, University of Toronto, Toronto, CAN; 3 General Dentistry and Implantology, Private Practice, Silver Spring, USA

**Keywords:** activated chlorine dioxide, antimicrobial mouth rinse, dental biofilm, gingival inflammation, gingivitis, oral hygiene, plaque control, toothbrushing

## Abstract

This prospective randomized study, examiner-blind, parallel-group clinical evaluating the comparative efficacy of a brush-and-rinse regimen versus a brush-and-floss regimen on plaque accumulation and gingival inflammation over 42 days. Seventy-six subjects brushed twice daily and were assigned to either daily flossing (control) or daily antimicrobial rinsing (test). Modified Gingival Index (MGI), Bleeding Index (BI), and Plaque Index (PI) were recorded at baseline, Day 14, and Day 42. The brush-and-rinse regimen demonstrated statistically significant reductions in MGI, BI, and PI from baseline (p<0.001). At Day 42, whole-mouth MGI improved 35% and BI 61.9%, while the control group showed minimal or negative change. Between-group analysis confirmed the statistical superiority of adjunctive rinsing for both gingival and plaque endpoints. Within the limitations of this six-week model, antimicrobial rinsing provided superior clinical outcomes compared with daily flossing.

## Introduction

Dental biofilm is universally recognized as the primary etiologic factor in the initiation and progression of gingivitis and periodontal disease [1]. Persistent supragingival plaque accumulation induces a localized inflammatory response characterized by vascular dilation, inflammatory cell infiltration, and clinical bleeding on probing [2]. Without adequate disruption, this inflammatory burden may predispose susceptible individuals to periodontal attachment loss.

Mechanical plaque removal via toothbrushing remains the cornerstone of preventive dentistry; however, interdental and marginal areas are frequently incompletely debrided [3]. Flossing has long been recommended as the primary interdental modality, yet its effectiveness is highly technique-dependent and often limited by patient compliance [4]. Clinical studies have demonstrated that toothbrushing combined with flossing provides greater reductions in interproximal plaque and gingival inflammation than toothbrushing alone. However, because daily flossing is not routinely performed by most patients, adjunctive mouthrinses have gained interest as an alternative approach. When used in conjunction with toothbrushing, antimicrobial mouthrinses have been shown to provide additional reductions in plaque accumulation, gingival inflammation, and bleeding compared with brushing alone. [5,6] Adjunctive chemotherapeutic agents, including antimicrobial mouthrinses, have therefore demonstrated additive benefits when incorporated into daily oral hygiene regimens [5,6].

Meta-analyses evaluating antiplaque and anti-gingivitis agents have consistently demonstrated statistically significant improvements in both clinical indices and bleeding parameters when antimicrobial rinses are used as adjuncts to brushing [5, 6]. The present randomized clinical trial was therefore designed to compare a brush-and-floss protocol with a brush-and-rinse regimen in a controlled six-week model, with the hypothesis that adjunctive rinsing would produce superior inflammatory and plaque outcomes.

Study objectives

The primary objective of this 42-day, randomized, examiner-blind clinical trial was to assess the efficacy and safety of a manual toothbrush with a test fluoride toothpaste (OraPro® dentifrice, OraCare, Bridgeport, WV, USA), followed by daily antimicrobial mouth rinsing, compared with a manual toothbrush with a marketed fluoride toothpaste, followed by daily dental flossing.

Primary endpoints included whole-mouth mean change in Modified Gingival Index (MGI) scores, Bleeding Index (BI) scores, and Turesky Plaque Index (TPI) scores at Day 42. The MGI provides a non-invasive assessment of gingival inflammatory severity [2]; the BI quantifies bleeding as an indicator of sulcular inflammation [7]; and the Turesky modification of the Quigley-Hein Plaque Index enables standardized plaque quantification [3].

Secondary endpoints included interim Day 14 changes and interproximal and marginal site analyses to evaluate early treatment effects and site-specific responses.

## Materials and methods

This study was conducted as a single-center, prospective, randomized, controlled, examiner-blind, parallel-group clinical study over 42 days in compliance with Good Clinical Practice (GCP) guidelines and applicable U.S. regulatory standards. Institutional Review Board approval was obtained from the U.S. Investigational Review Board, Inc. (approval number: U.S.IRB2025SRI/08) to conduct this study, and all participants provided written informed consent prior to enrollment.

Study population

Approximately 80 generally healthy adults (≥18 years) were screened to achieve 70 evaluable subjects (35 per group). Inclusion criteria required ≥18 natural teeth and baseline gingival inflammation (MGI ≥1.75), plaque accumulation (TPI ≥1.95 following 12-18-hour plaque accumulation), and ≥10 bleeding sites (BI). Key exclusion criteria included active periodontal therapy, recent antibiotic or anti-inflammatory use, tobacco use, orthodontic appliances, significant dental pathology, or systemic conditions that could affect study outcomes. Clinical indices recorded at baseline, Day 14, and Day 42 included the MGI [2], BI [7], and TPI [3, 8]. Statistical analysis was performed using ANOVA and appropriate comparative testing to assess both within-group and between-group differences. Each study patient underwent a professional dental prophylaxis prior to initiation of their participation in the study.

Study design and interventions

Eligible subjects were randomized (1:1) using a sponsor-generated allocation schedule and stratified by baseline bleeding scores. Examiners were blinded to treatment assignment. Patients were given oral hygiene instructions, part of which was a brushing technique, but as patients typically fall back into their own brushing techniques, the study evaluated results over different patients' usual brushing techniques.

Control Group

**Figure 1:**
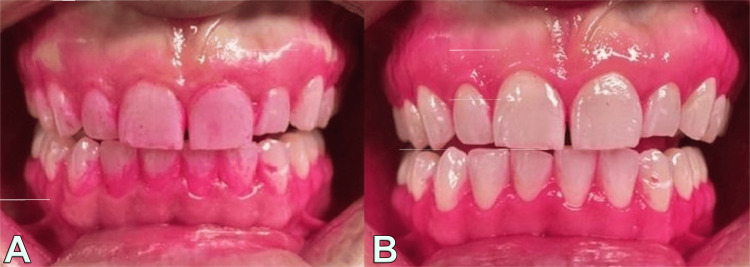
Plaque accumulation and its associated bacteria in the morning following use of disclosing solution (A) and after use of toothbrushing with a micro-netting technology toothpaste and an activated chlorine dioxide adjunctive antimicrobial rinse (B).

Twice-daily brushing with a flat-trim manual toothbrush (Oral-B Indicator 35, Oral-B Laboratories, Iowa City, IA, USA) and fluoride dentifrice (Crest® Cavity Protection, Procter & Gamble, Cincinnati, OH, USA), followed by once-daily flossing (Oral-B Glide, Oral-B Laboratories) (Figure 1A).

Test Group

Twice-daily brushing with the same toothbrush and a micro-netting technology dentifrice (OraPro®), utilizing engineered silica particles designed to enhance plaque and biofilm disruption, followed by twice-daily rinsing with an activated chlorine dioxide mouth rinse (OraCare Health Rinse, OraCare) (Figure 1B).

All subjects brushed for two minutes per session and were instructed to avoid all non-study oral hygiene products during the trial. Initial use of assigned regimens was supervised at baseline.

Clinical assessments

Clinical evaluations were performed at baseline, Day 14 (±2 days), and Day 42 (±2 days). Subjects refrained from oral hygiene for 12-18 hours and from eating for 30 minutes prior to each visit.

Primary clinical endpoints included the following: MGI: assessed on buccal and lingual gingival units (score 0-4); BI: assessed following gentle probing (score 0-2); TPI: evaluated using disclosed plaque on six surfaces per tooth.

Whole-mouth mean scores were calculated, with additional analyses of interproximal and gingival margin plaque.

Compliance and safety

Compliance was monitored through subject diaries and by weighing returned study materials at follow-up visits. Safety assessments included intraoral examinations and documentation of adverse events at each visit, categorized by severity and relationship to study products.

Examiner calibration

Four examiners were trained and calibrated prior to study initiation, achieving ≥80% agreement in repeatability assessments for MGI and TPI scoring.

Statistical analysis

A sample size of 70 evaluable subjects provided 90% power to detect differences between groups at Day 42 (α=0.05), assuming effect sizes of 0.24 (MGI) and 0.4 (TPI). Descriptive statistics were calculated for all variables. Within- and between-group comparisons were analyzed using ANOVA with two-sided significance testing (p<0.05).

## Results

Study population

**Figure 2:**
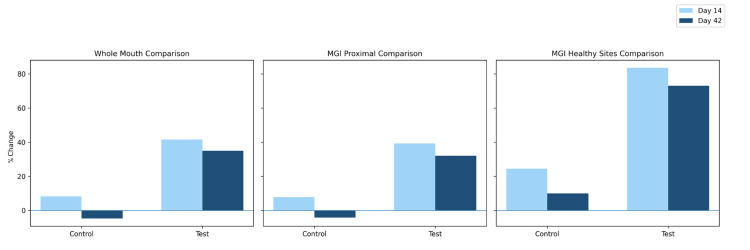
Change (% improvement) of healthy sites measured by the Modified Gingival Index (MGI).

**Figure 3:**
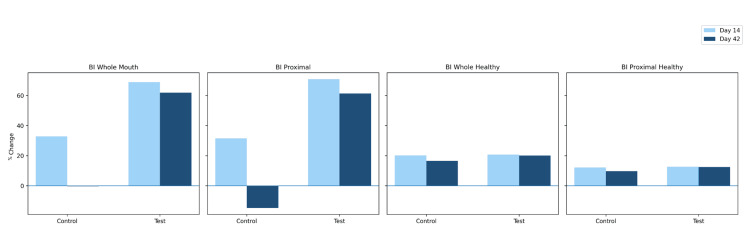
Change (% improvement) measured by the Bleeding Index (BI).

**Figure 4:**
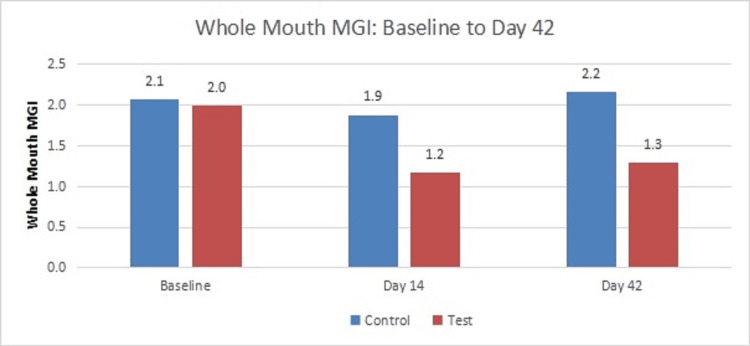
Comparison of whole-mouth outcomes between the control and test groups from baseline to day 42 MGI: Modified Gingival Index

Seventy-six subjects were randomized, and 71 completed the study protocol. Five patients failed to complete the study by following through on scheduled appointments and were not included in the final data. Demographic characteristics for enrolled and evaluable subjects are detailed in Tables 1-2, respectively. Baseline age, gender distribution, and race were comparable between groups, with no statistically significant differences in age (p>0.90) (Figures 2-3). All participants were non-smokers. Although ethnicity distribution differed between groups in the evaluable population (Table 1), baseline gingival health status, as measured by whole-mouth healthy MGI site counts, was not statistically different (p=0.252), confirming appropriate randomization and comparable inflammatory status at study initiation (Figure 4).

**Table 1:** Demographic characteristics of the enrolled subjects ^1^p-value for age, p=0.933, from an ANOVA; the other p-values from a Fisher’s exact test: gender p=0.628, race p=0.371, and ethnicity p=0.021.

Variable	Control (N=38)	Test (N=38)	Total (N=76)	P value^1^
Age (years)				0.933
N	38	38	76	
Mean ± SD	51.2 ± 16.10	49.7 ± 14.30	50.5	
Standard Error	2.61	2.32	1.74	
Minimum	20	18	18	
Maximum	79	80	80	
Gender				0.628
Male n(%)	4 ( 10.5%)	7 ( 18.4%)	11 ( 14.5%)	
Female n(%)	34 ( 89.5%)	31 ( 81.6%)	65 ( 85.5%)	
Race				0.371
American Indian/Alaskan Native n(%)	1 (2.6%)	0 (0.0%)	1 (1.3%)	
Black/African American n(%)	2 (5.3%)	4 (10.5%)	6 (7.9%)	
White n(%)	32 (84.2%)	34 (89.5%)	66 (86.8%)	
Asian n(%)	2 (5.3%)	0 (0.0%)	2 (2.6%)	
Other n(%)	1 (2.6%)	0 (0.0%)	1 (1.3%)	
Ethnicity				0.021
Hispanic/Latino n(%)	0 (0.0%)	5 (13.2%)	5 (7.0%)	
White/Black/Asian n(%)	38 (100.0%)	33 (86.8%)		
Tobacco Use				
Non-smoker n(%)	38 (100.0%)	38 (100.0%)		

**Table 2:** Demographic sharacteristics of the evaluable subjects ^1^p-value for age p=0.938 from an ANOVA; the other p-values from a Fisher’s exact test: gender p=0.540, race p=0.675, and ethnicity p=0.021.

	Control (N=37)	Test (N=34)	Total (N=71)	P-value^1^
Age (years)				0.938
N	37	34	71	
Mean	51.8	50.2	51.1	
Standard deviation	15.81	14.85	15.27	
Standard error	2.6	2.55	1.81	
Minimum	20	18	18	
Maximum	79	80	80	
Gender				0.54
Male	4 (10.8%)	7 (20.6%)	11 (15.5%)	
Female	33 (89.2%)	27 (79.4%)	60 (84.5%)	
Race				0.675
American Indian/Alaskan Native	1 (2.7%)	0 (0.0%)	1 (1.4%)	
Black/African American	2 (5.4%)	3 (8.8%)	5 (7.0%)	
White	31 (83.8%)	31 (91.2%)	62 (87.3%)	
Asian	2 (5.4%)	0 (0.0%)	2 (2.8%)	
Other	1 (2.7%)	0 (0.0%)	1 (1.4%)	
Ethnicity				0.021
Hispanic/Latino	0 (0.0%)	5 (14.7%)	5 (7.0%)	
White/Black/Asian	37 (100.0%)	29 (85.3%)	66 (93.0%)	
Tobacco Use				
Non-smoker	37 (100.0%)	34 (100.0%)	71 (100.0%)	

Modified Gingival Index

Day 14

**Figure 5:**
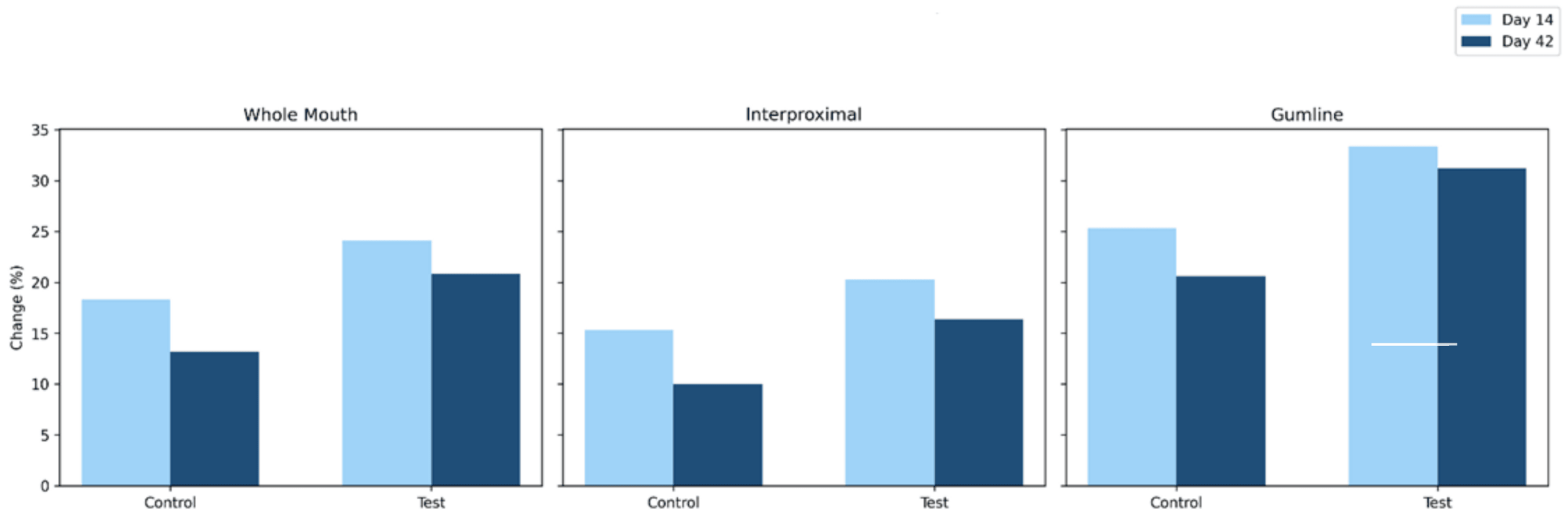
Modified Gingival Index summary statistics

Interim results at Day 14 showed statistically significant improvements in both groups; however, the magnitude of improvement was substantially greater in the test cohort (Figure 5).

**Figure 6:**
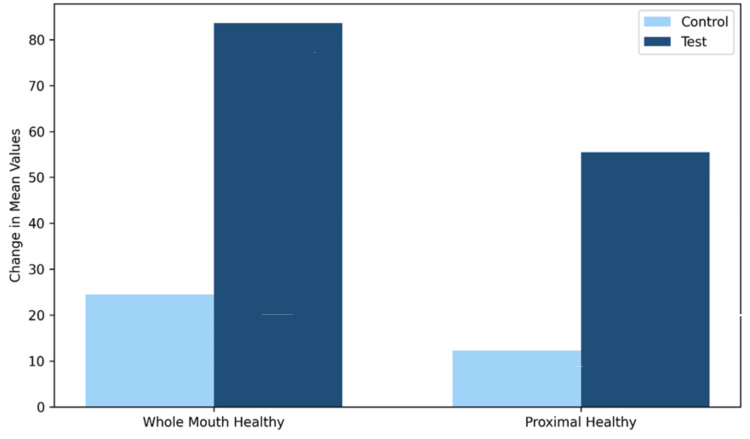
Healthy site changes (Day 14).

Whole-mouth MGI improved by 0.83 in the test group compared with 0.18 in the control group (p < 0.001 for both within-group changes). Between-group ANOVA analysis confirmed the superiority of the test regimen at Day 14 (Figure 6; p < 0.001).

**Figure 7:**
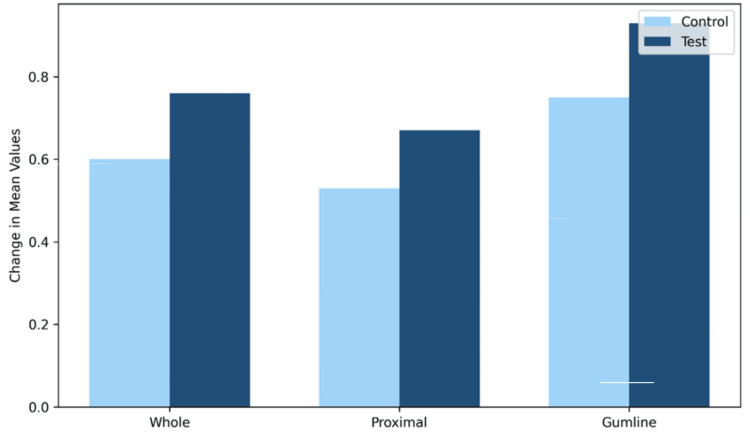
Plaque changes (Day 14).

Change in number of healthy MGI sites at Day 14 also favored the test group, with statistically significant between-group differences for both whole-mouth and interproximal sites (Figure 7; p<0.001).

Day 42

**Figure 8:**
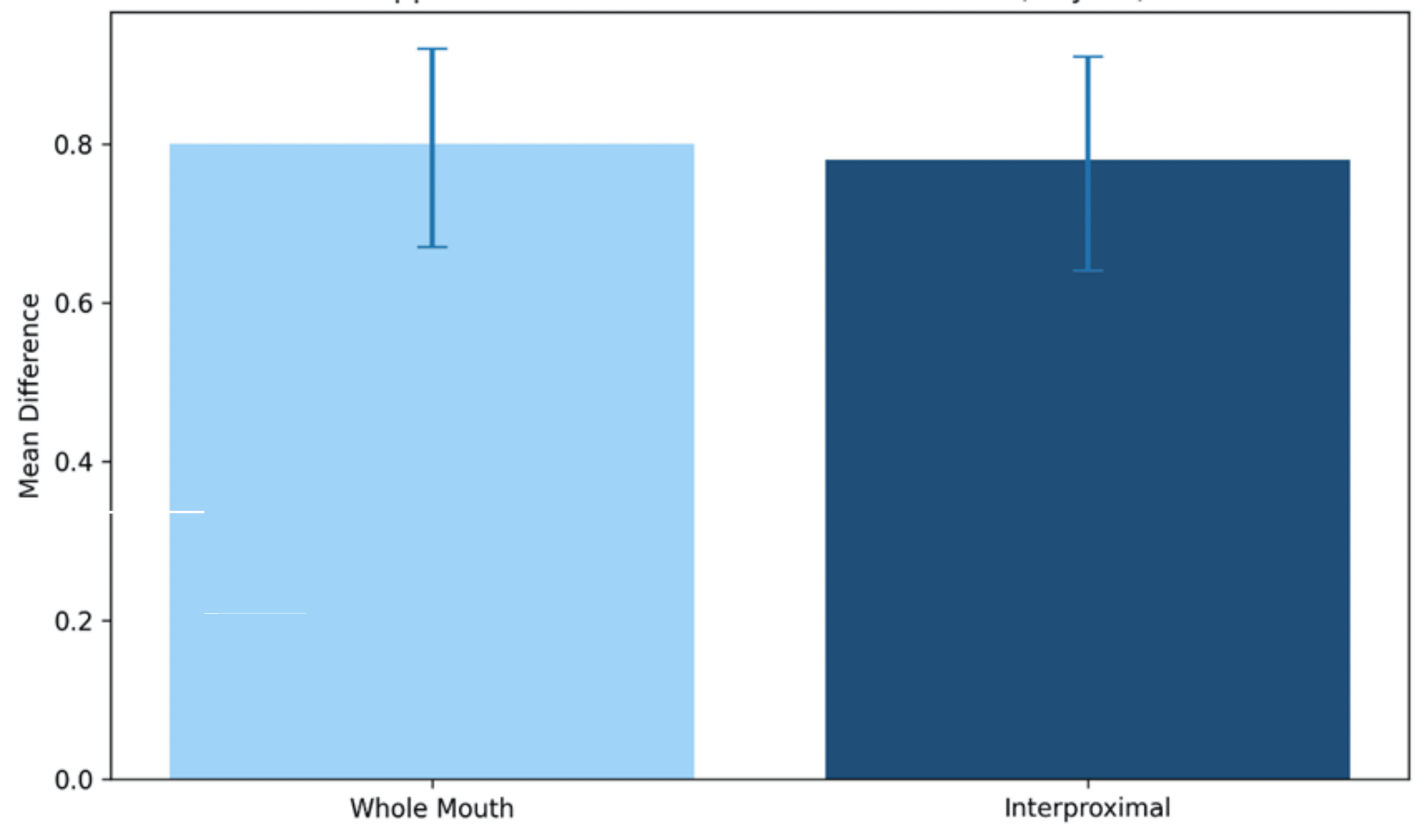
ANOVA results for Modified Gingival Index (Day 42).

At Day 42, the test group demonstrated statistically significant reductions in whole-mouth and interproximal MGI compared with baseline (p<0.001), whereas the control group showed slight regression from baseline (Figure 8).

In the control group, mean whole-mouth MGI increased from 2.07 at baseline to 2.16 at Day 42, reflecting a negative change of −0.10 (p=0.007). Interproximal MGI similarly regressed (−0.09; p=0.014).

In contrast, the test group demonstrated a mean whole-mouth MGI reduction from 2.00 to 1.30, representing a change of 0.70 (p<0.001). Interproximal MGI improved by 0.68 (p<0.001).

**Figure 9:**
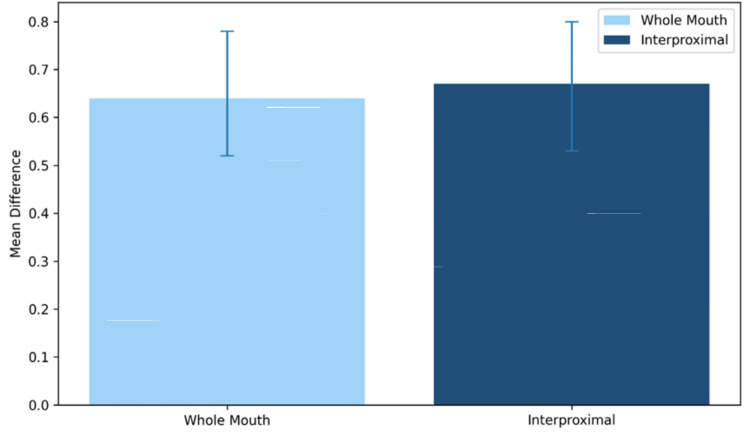
ANOVA results for Modified Gingival Index (Day 42).

Between-group ANOVA analysis confirmed statistically significant superiority of the test regimen for both whole-mouth and interproximal MGI at Day 42 (Figure 9; p<0.001 for both comparisons).

**Figure 10:**
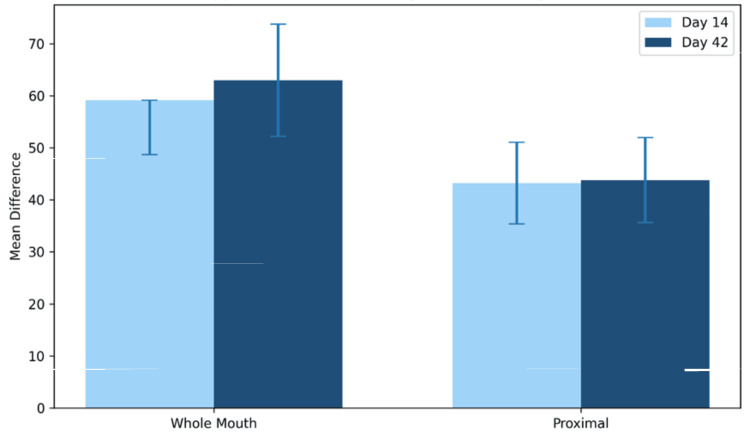
Change in Modified Gingival Index healthy sites.

Change to a healthy gingival status further emphasized clinical relevance. At Day 42, the mean change in the number of whole-mouth healthy sites was 72.98 in the test group compared with 9.97 in the control group. The between-group difference was highly significant (Figure 9; p<0.001), with the magnitude of improvement in the test group approximately seven times greater than that of the control group (Figure 10)

Bleeding Index

Day 14

**Figure 11:**
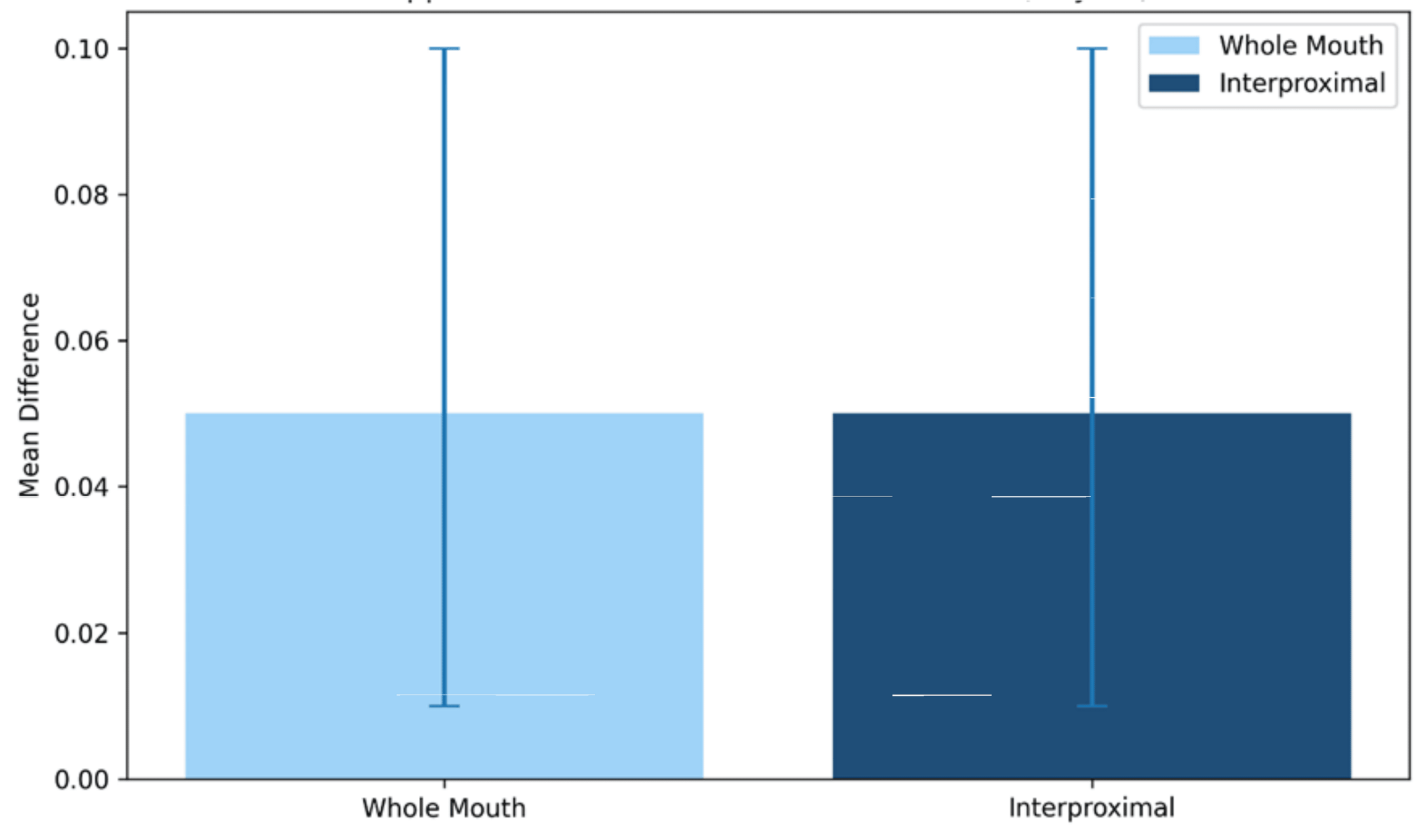
ANOVA results for Bleeding Index (Day 14).

At Day 14, both groups demonstrated statistically significant reductions in BI from baseline (Figure 11). However, the magnitude of reduction was greater in the test group.

Between-group ANOVA results demonstrated statistical superiority for the test regimen at Day 14 for whole-mouth and interproximal BI (Figure 11; p=0.018 and p=0.017, respectively).

Day 42

**Figure 12:**
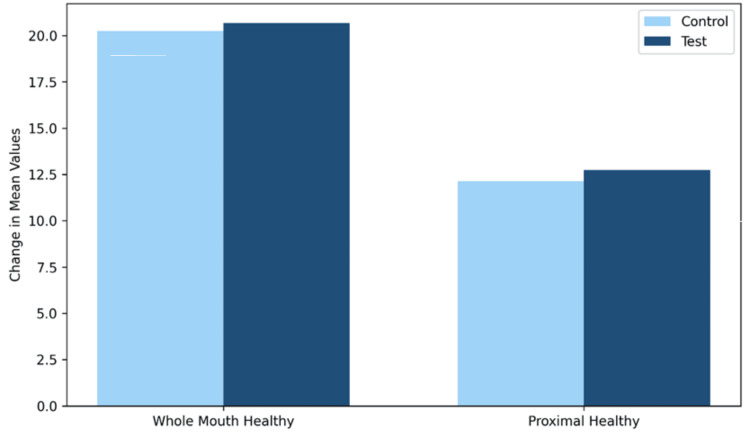
Healthy site changes (Day 42).

BI results paralleled MGI findings. At Day 42, the control group demonstrated no statistically significant improvement in whole-mouth BI (change −0.00; p=0.968), and interproximal BI change did not reach significance (p=0.140) (Figure 12).

Conversely, the test group demonstrated a mean whole-mouth BI reduction of 0.11 and an interproximal reduction of 0.10 (p<0.001 for both comparisons).

**Figure 13:**
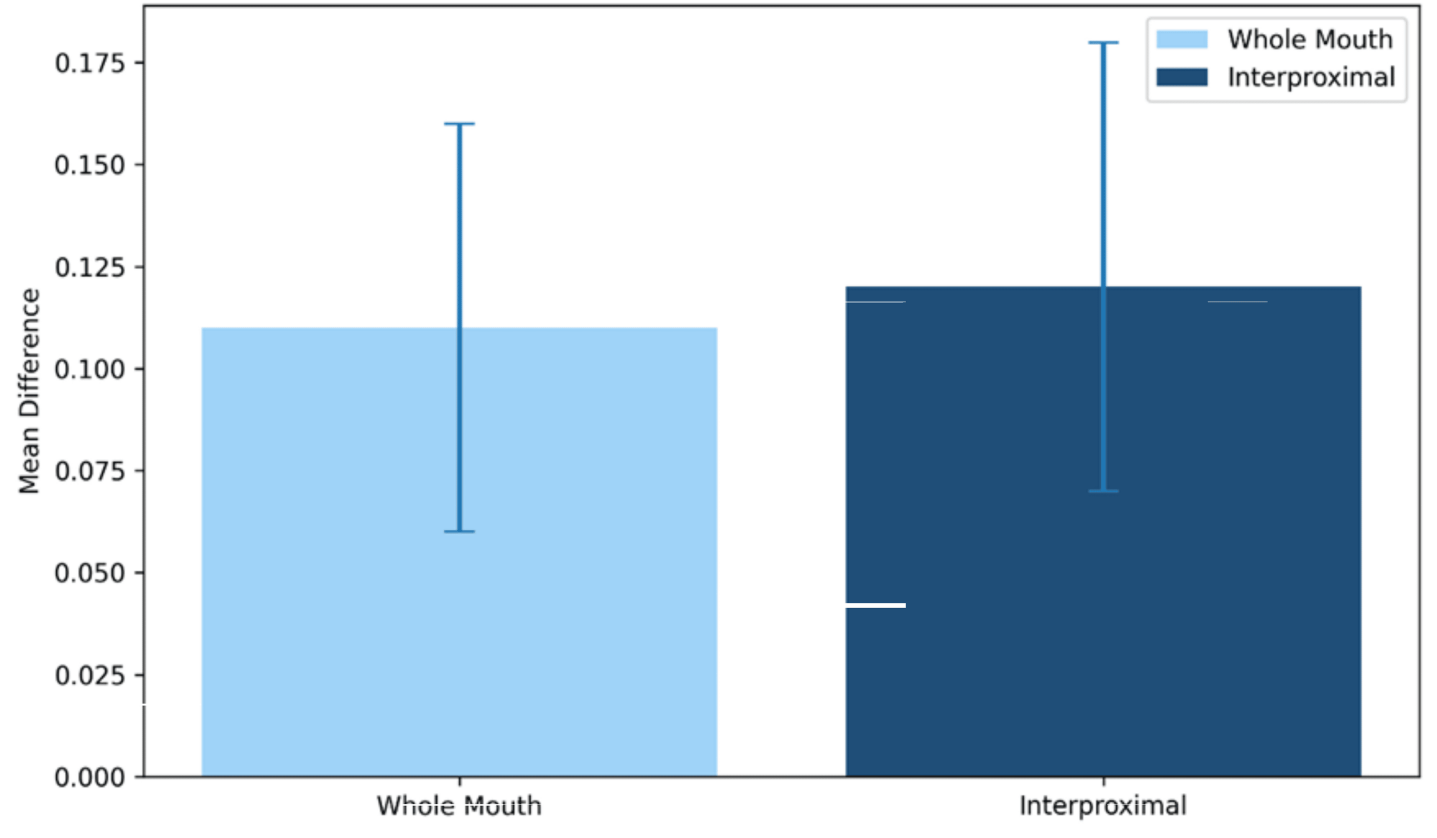
ANOVA results for Bleeding Index (Day 42).

ANOVA analysis confirmed statistically significant superiority of the test regimen over control for both whole-mouth and interproximal BI at Day 42 (Figure 13; p<0.001).

**Figure 14:**
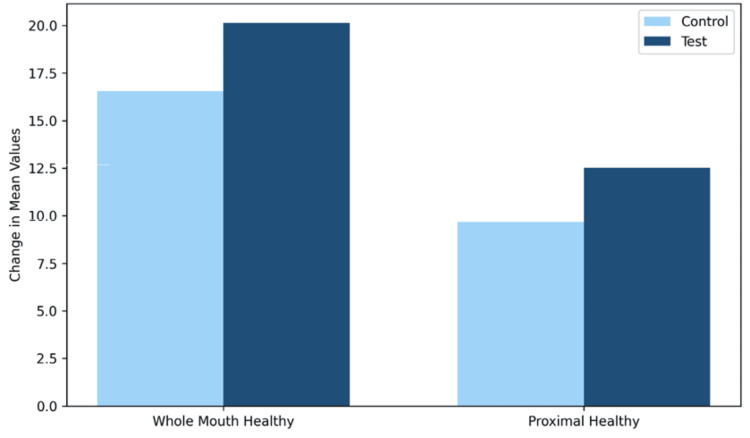
Healthy site changes (Day 42).

**Figure 15:**
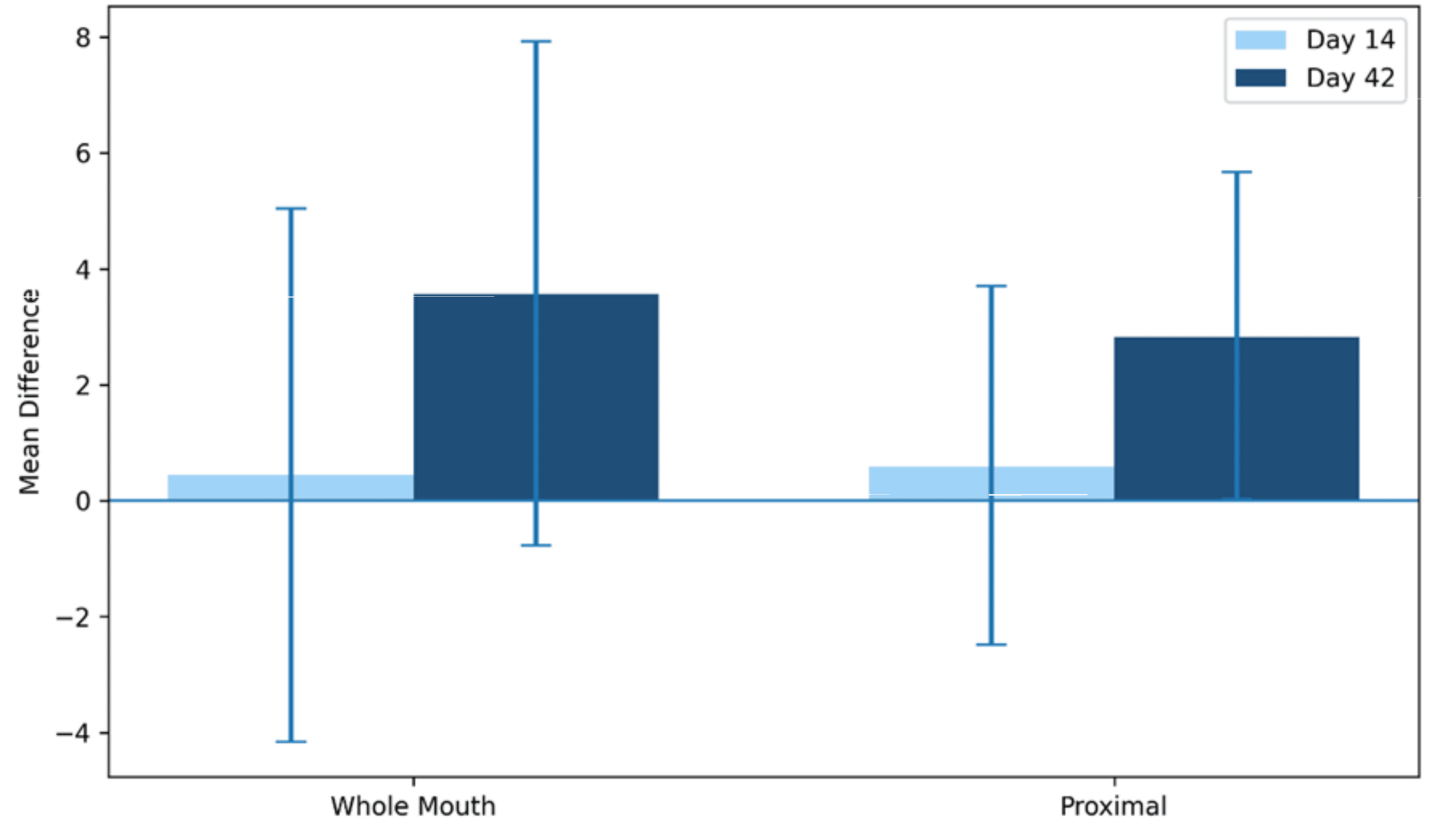
Healthy bleeding site changes.

Change to a healthy bleeding status further supported these findings. The test group demonstrated greater improvement in the mean number of healthy bleeding sites compared with the control; however, differentiation for some site categories did not reach statistical significance (Figures 14-15).

Turesky Plaque Index

Day 14

**Figure 16:**
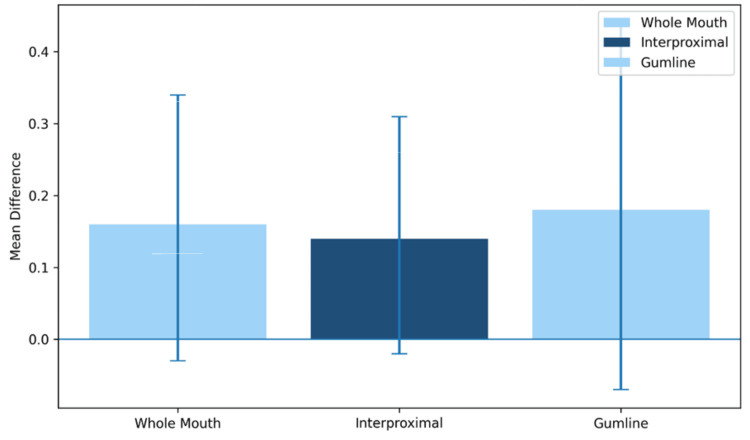
ANOVA results for plaque (Day 14).

At Day 14, both groups demonstrated significant reductions from baseline across plaque parameters. The test group exhibited numerically greater reductions; however, between-group differences did not reach statistical significance at this interim evaluation (Figure 16; p>0.05).

This pattern suggests a cumulative chemotherapeutic effect, with greater differentiation between regimens becoming evident by Day 42.

Day 42

**Figure 17:**
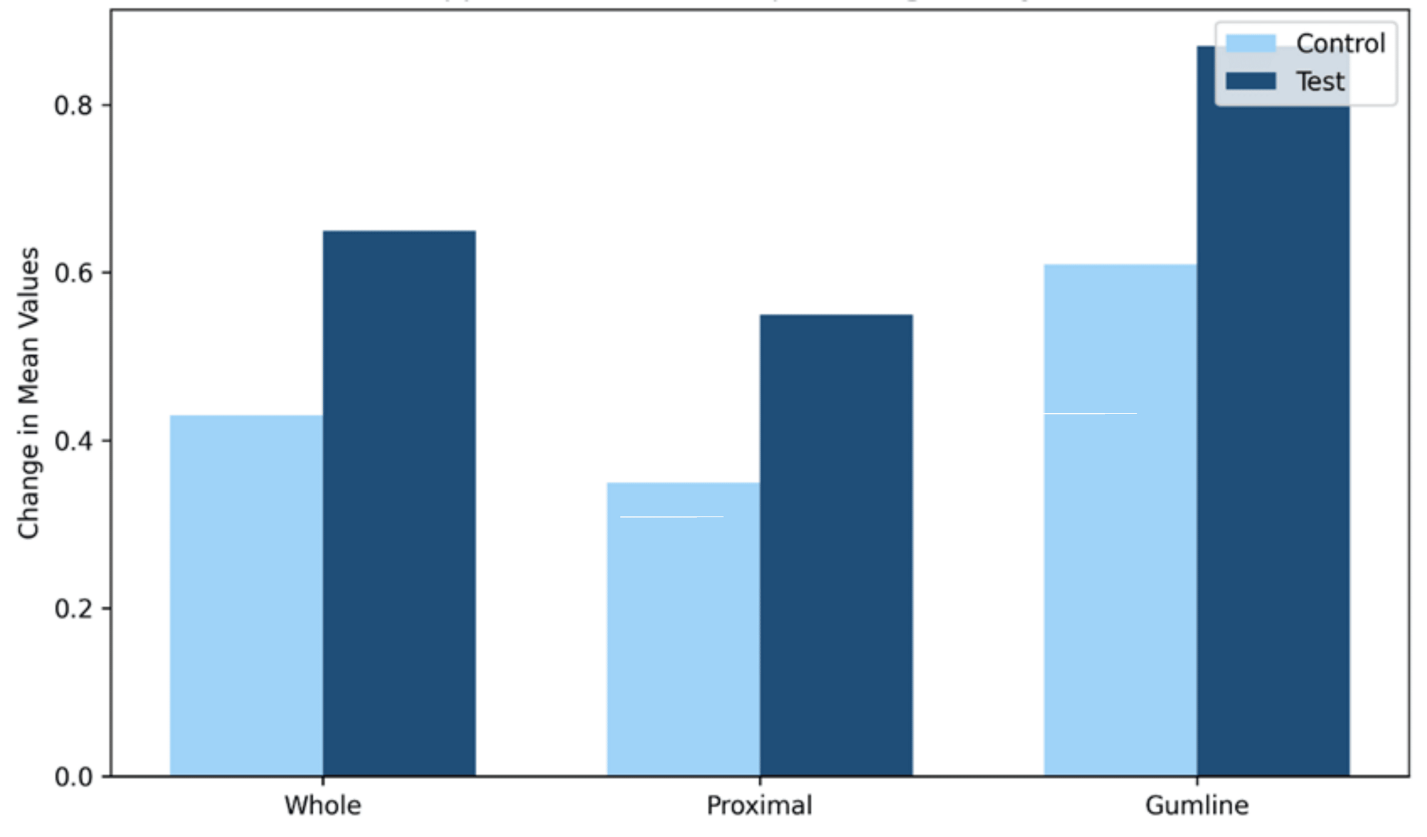
Plaque changes (Day 42).

Plaque reduction results are summarized in Figure 17. Both groups demonstrated statistically significant reductions from baseline (p<0.001).

In the control group, whole-mouth plaque decreased by 0.43, interproximal by 0.35, and gumline by 0.61. In the test group, reductions were greater: 0.65 for whole-mouth, 0.55 for interproximal, and 0.87 for gumline sites.

**Figure 18:**
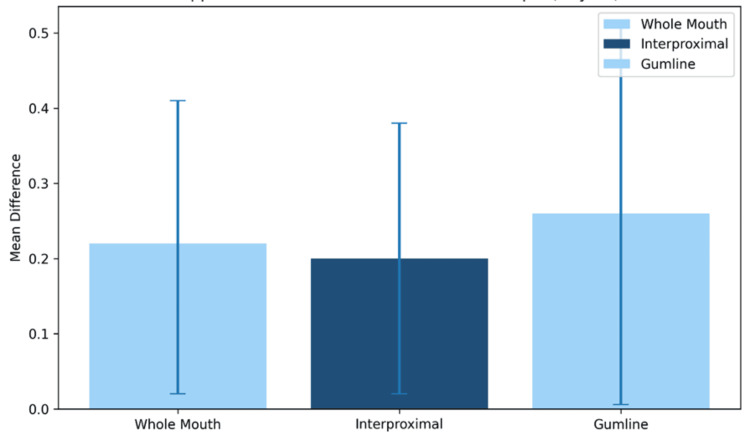
ANOVA results for plaque (Day 42).

Between-group ANOVA analysis confirmed statistically significant superiority of the test regimen at Day 42 for whole-mouth (p=0.028), interproximal (p=0.027), and gumline plaque (p=0.045) (Figure 18).

These findings demonstrate that plaque reduction in the test cohort was approximately 50% greater than that observed in the control group at the final evaluation.

Safety

Oral soft tissue examinations revealed no treatment-related adverse events. Two mild events unrelated to study regimens were reported, including localized cheek trauma and thermal irritation from food. No clinically significant mucosal or gingival abnormalities were observed throughout the study period.

OraCare dentifrice and mouthrinse combination

The comparison between mouthrinsing and flossing is clinically relevant because both are commonly recommended adjuncts to toothbrushing. However, because daily flossing is not routinely performed by most patients, this study evaluated whether adding a therapeutic mouthrinse in place of flossing could improve plaque control while offering a simpler, less technique-sensitive approach to oral hygiene.

The demonstrated superiority of a brush-and-rinse regimen over brush-and-floss protocols supports the clinical relevance of integrated chemotherapeutic systems such as the OraCare dentifrice and mouthrinse combination. Utilizing activated chlorine dioxide technology, this system provides both mechanical and chemical disruption of oral biofilm while neutralizing bacterial byproducts associated with gingival inflammation.

During brushing, the dentifrice facilitates initial biofilm disruption, while subsequent rinsing extends antimicrobial activity into interproximal and marginal regions that are less accessible to mechanical instrumentation. This sequential approach is consistent with the findings of the present study, where adjunctive rinsing produced significantly greater reductions in MGI, BI, and Plaque Index compared with flossing alone.

Clinically, this enhanced biofilm control translates to improved gingival stability, reduced bleeding, and simplified patient compliance. The absence of alcohol and a favorable safety profile further support routine daily use without adverse tissue effects, consistent with the lack of treatment-related events observed in this trial.

Incorporation of a dentifrice-rinse combination such as OraCare into daily oral hygiene protocols offers a practical, compliance-friendly adjunct to mechanical plaque removal, with the potential to enhance inflammatory control and improve overall clinical outcomes.

## Discussion

The results of this 42-day randomized, examiner-blind clinical trial demonstrate that adjunctive antimicrobial rinsing following twice-daily toothbrushing provides statistically and clinically significant improvements in gingival health and plaque reduction when compared with daily flossing. The magnitude and consistency of these findings across primary inflammatory and plaque endpoints at Day 42 suggest both immediate antimicrobial effects and a cumulative therapeutic benefit over time. The progressive divergence between groups from Day 14 to Day 42 further supports a sustained chemotherapeutic influence that enhances biofilm control beyond mechanical disruption alone.

Gingival inflammation represents a host-mediated response to persistent microbial biofilm and remains the initiating factor in the pathogenesis of periodontal disease. Foundational experimental gingivitis studies have clearly demonstrated a direct, causative relationship between plaque accumulation and gingival inflammation, with resolution occurring following effective biofilm removal [9]. Bleeding on probing, reflected in this study by the BI, is widely regarded as a sensitive and reproducible indicator of gingival inflammation and a predictive marker for periodontal stability or disease progression [10, 11]. The greater than 60% reduction in whole-mouth BI observed in the rinse cohort is therefore clinically meaningful and indicative of a substantial reduction in inflammatory burden. This degree of improvement suggests not only suppression of microbial challenge but also modulation of the host inflammatory response at the gingival interface.

The MGI findings further corroborate these observations. Statistically significant reductions in both whole-mouth and interproximal MGI scores, coupled with a higher proportion of sites converting to clinically healthy status, reflect meaningful resolution of gingival inflammation. Importantly, such site-level transitions from inflamed to non-inflamed states are of greater clinical relevance than mean score reductions alone, as they represent restoration of tissue homeostasis and reduced susceptibility to future periodontal breakdown.

Interproximal plaque control remains one of the most challenging aspects of daily oral hygiene. Although dental floss has long been advocated as the standard of care for interproximal cleaning, its clinical effectiveness is highly dependent on patient technique, manual dexterity, and compliance. Systematic reviews and meta-analyses have reported limited and inconsistent additional benefit of flossing over toothbrushing alone in populations with variable adherence [4, 12]. In contrast, antimicrobial mouth rinses have demonstrated consistent adjunctive benefits in reducing both plaque accumulation and gingival inflammation when used in conjunction with mechanical plaque control [6, 13, 14]. The findings of the present study align with this body of evidence, particularly in demonstrating enhanced interproximal plaque suppression in the rinse cohort, an area traditionally resistant to effective mechanical cleaning.

From a biological perspective, the observed improvements are plausible given the ability of antimicrobial rinses to penetrate areas that are less accessible to mechanical devices, including gingival margins, shallow sulcular environments, and interproximal niches. Chemotherapeutic agents can disrupt bacterial cell walls, inhibit biofilm maturation, and reduce overall microbial load, thereby limiting the ecological conditions that favor pathogenic biofilm development [15]. This adjunctive effect is particularly relevant in early gingivitis management, where reversible inflammatory changes can be effectively controlled through reduction of microbial burden.

The absence of treatment-related adverse events in this study supports the short-term safety and tolerability of daily antimicrobial rinsing. Clinically, improved gingival stability may have broader implications beyond periodontal health, including reduced bleeding during restorative and surgical procedures, improved visualization during treatment, and enhanced soft tissue integration around restorative margins and implant-supported prostheses. These factors contribute to both procedural efficiency and long-term maintenance of oral health.

The clinical implications of these findings are particularly relevant in the context of patient compliance. While flossing remains an important component of oral hygiene, real-world adherence is often suboptimal. The incorporation of an adjunctive antimicrobial rinse may provide a more accessible and less technique-sensitive alternative or supplement, thereby improving the overall effectiveness of home care regimens. This is consistent with contemporary preventive strategies that emphasize simplification of patient routines to enhance compliance and clinical outcomes.

Several limitations should be considered when interpreting these results. Patient compliance with prescribed oral hygiene protocols, including both brushing and rinsing, could not be directly verified and may have influenced outcomes. Additionally, the relatively short duration of the study (42 days) limits extrapolation to long-term periodontal stability and disease prevention. Future studies with extended follow-up periods, larger sample sizes, and microbiologic or biomarker analyses would provide further insight into the sustained effects and mechanisms of adjunctive antimicrobial therapy.

Within these limitations, the consistent and statistically significant improvements observed across multiple clinical parameters support the incorporation of adjunctive antimicrobial rinsing as an effective enhancement to daily oral hygiene protocols. This approach may be particularly beneficial in patients with suboptimal flossing compliance or those at increased risk for gingival inflammation and early periodontal disease.

## Conclusions

Within the limitations of this 42-day randomized, examiner-blind clinical trial, the use of an activated chlorine dioxide antimicrobial rinse following twice-daily toothbrushing with a micro-netting technology dentifrice was associated with statistically significant improvements in gingival inflammation, bleeding, and plaque accumulation compared with the brush-and-floss regimen evaluated. The greater reductions in BI and MGI scores observed in the rinse cohort suggest that adjunctive antimicrobial rinsing may provide additional benefits for controlling supragingival biofilm and gingival inflammation.

The progressive separation between groups from Day 14 to Day 42 may indicate a cumulative benefit associated with the rinse-based regimen; however, the relatively short study duration and specific products evaluated limit extrapolation of these findings to long-term periodontal outcomes or to other oral hygiene products. While flossing remains an established method of interproximal plaque removal, patient compliance and technique variability may influence its clinical effectiveness. Within the parameters of this study, adjunctive antimicrobial rinsing represented a practical alternative that achieved favorable clinical outcomes. Additional long-term studies are warranted to determine the durability of these effects and their impact on periodontal health in broader patient populations.
